# Optimization and analytical validation of the Allplex HPV28 genotyping assay for use in first-void urine samples

**DOI:** 10.1128/jcm.01404-24

**Published:** 2024-12-26

**Authors:** Margo Bell, Iacopo Baussano, MaryLuz Rol, Vanessa Tenet, Daniëlle A. M. Heideman, Tarik Gheit, Anne Van Caesbroeck, Alex Vorsters, Gary Clifford

**Affiliations:** 1 Centre for the Evaluation of Vaccination (CEV), Vaccine & Infectious Disease Institute (VAXINFECTIO), Faculty of Medicine and Health Sciences, University of Antwerp 198686 , Antwerp, Flanders, Belgium; 2 International Agency for Research on Cancer 56140 , Lyon, France; 3 Department of Pathology and Medical Biology, University Medical Center Groningen, University of Groningen 3647 , Groningen, Groningen, the Netherlands; St. Jude Children's Research Hospital, Memphis, Tennessee, USA

**Keywords:** first-void urine, self-sampling, HPV genotyping, vaccine impact monitoring

## Abstract

Despite first-void urine (FVU) being increasingly recognized as a credible specimen for human papillomavirus (HPV) detection, there is a lack of well-validated testing methods providing full quantitative genotyping required for vaccine impact monitoring from FVU samples. The Allplex HPV28 assay, capable of individually detecting 28 HPV genotypes, presents a promising method. We aimed to evaluate its genotype-specific performance on FVU samples, following optimization of FVU preanalytics. We selected 701 FVU samples collected using a Colli-Pee device (20 mL, with UCM), enriched for HPV-positivity (*n* = 630) based on previous testing with GP5+/6+-PCR-based reverse line blot (GP5+/6+ RLB) and E7-MPG after Amicon filtration (AF). We first evaluated the comparability and agreement of Allplex HPV28 genotype-specific positivity according to different preanalytics. Subsequently, we conducted the genotype-specific comparison of Allplex HPV28 with GP5+/6+ RLB AF and E7-MPG AF. No significant differences in HPV positivity by Allplex HPV28 testing were observed when comparing pre-centrifuged versus non-centrifuged DNA extraction, nor when comparing manual versus automated DNA extraction. Good genotype-specific agreement was observed between Allplex HPV28 and GP5+/6+ RLB AF, with Allplex HPV28 being slightly more sensitive for all 28 HPV genotypes (average Allplex HPV28:GP5+/6+ RLB AF ratio 1.729). Compared to E7-MPG AF, Allplex HPV28 exhibited lower sensitivity for all 21 overlapping HPV genotypes (average Allplex HPV28:E7-MPG AF ratio 0.588). The findings of this study, combined with practical considerations for real-world implementation, support the use of Allplex HPV28 testing after automated or manual DNA extraction without the requirement for pre-centrifugation, for HPV studies based on FVU samples, most notably those for vaccine impact monitoring on HPV prevalence.

**IMPORTANCE:**

This study provides the first analytical validation of the Allplex HPV28 genotyping assay for use in first-void urine samples, offering a reliable, non-invasive, and practical alternative to cervical samples for human papillomavirus (HPV) detection. It demonstrates a validated approach that supports the assay’s potential application in real-world settings, including low- and middle-income countries, where non-invasive and widely acceptable sampling methods are crucial for maximizing population coverage and representativity. Given the urgent need for accurate and practical tools to monitor HPV vaccination impact, these findings offer a timely and impactful contribution to the field.

## INTRODUCTION

First-void urine (FVU) is increasingly recognized as a valid, non-invasive, and user-friendly alternative for detecting female cervical human papillomavirus (HPV) infection, both for cervical cancer screening (1–9) and for epidemiological surveys of HPV prevalence, most notably to assess HPV vaccine effectiveness (10–14). The rationale behind the use of FVU for HPV detection is based on the fact that during urination, the first part of the urine captures mucus and debris from exfoliated cells from the female genital organs (15, 16). Numerous studies have demonstrated strong correlation between urinary and cervical HPV DNA detection (1, 17–23).

Evaluations of the efficacy and effectiveness of HPV vaccination rely upon genotype-specific detection of HPV infections, more specifically in sentinel surveys of adolescent girls and young women. The International Agency for Research on Cancer (IARC) has pioneered population-based surveys evaluating HPV vaccination impact in multiple low- and middle-income countries (LMIC) (11, 13, 24), and has established the utility of FVU self-sampling (13, 14). Indeed, surveys conducted in early vaccine introducing LMIC of Rwanda and Bhutan showed that 94% of young women returned self-collected urine samples for HPV testing, proving high acceptance, regardless of their sexual activity status, and maximizing population representativity (11). Moreover, multiple studies have demonstrated FVU to be preferred over gynecological examination or self-collection of vaginal samples (2, 9, 25–27).

HPV detection and genotyping in past IARC FVU surveys were performed using the GP5+/6+ PCR-enzyme immunoassay (EIA), followed by reverse line blot hybridization (GP5+/6+ RLB) (11, 13–15, 28), a well-validated test long considered as the standard comparator for evaluating new cervical cancer screening tests (29). However, this assay and its genotyping RLB readout are based on technologies that are not in widespread use anymore. This underscores the need for validating alternative methods for HPV genotype-specific testing in FVU samples. In this context, the Allplex HPV28 assay (Seegene Inc., South Korea) presents a promising solution. With its capability to individually detect 28 HPV genotypes utilizing an open and automated platform, it holds significant potential for epidemiological studies and vaccine effectiveness evaluations.

To date, the Allplex HPV28 assay has been validated for cervical and self-collected vaginal specimens (30, 31). The aim of our study was to evaluate the genotype-specific performance of the Allplex HPV28 assay on FVU samples and to optimize and simplify FVU preanalytics. Toward this goal, we performed an extensive HPV genotype-specific comparison of Allplex HPV28 in FVU samples collected from IARC surveys, which had been previously tested with both GP5+/6+ RLB and the highly analytically sensitive E7-MPG assay (15).

## MATERIALS AND METHODS

### FVU sample collection and selection

FVU samples were collected from 2014 to 2019 in the framework of HPV vaccine effectiveness studies in Rwanda, Bhutan, and Armenia, coordinated by the IARC. Sample collection methods and population characteristics of the surveys are reported elsewhere (11, 13). Briefly, FVU samples were self-collected using a 20 mL Colli-Pee device with Urine Conservation Medium (UCM) (Novosanis, Belgium) in the respective countries, and were shipped and stored at −80°C until analysis. HPV testing was performed both by GP5+/6+ RLB and E7-MPG (11, 13), following DNA extraction at the University of Antwerp, Belgium, between 2016 and 2020 (see below). All included studies had approval from both the local Research Ethical Boards and the IARC Ethics Committee and all participants provided written informed consent.

A total of 701 FVU samples were selected, including all available positives for any HPV genotype by E7-MPG and/or GP5+/6+ RLB, leading to a diverse assortment of the different high-risk (defined as IARC Group I) and other HPV genotypes (*n* = 630), as well as an additional ~10% of samples negative by both HPV tests, selected randomly (*n* = 71).

### Sample testing flow

**Fig 1:**
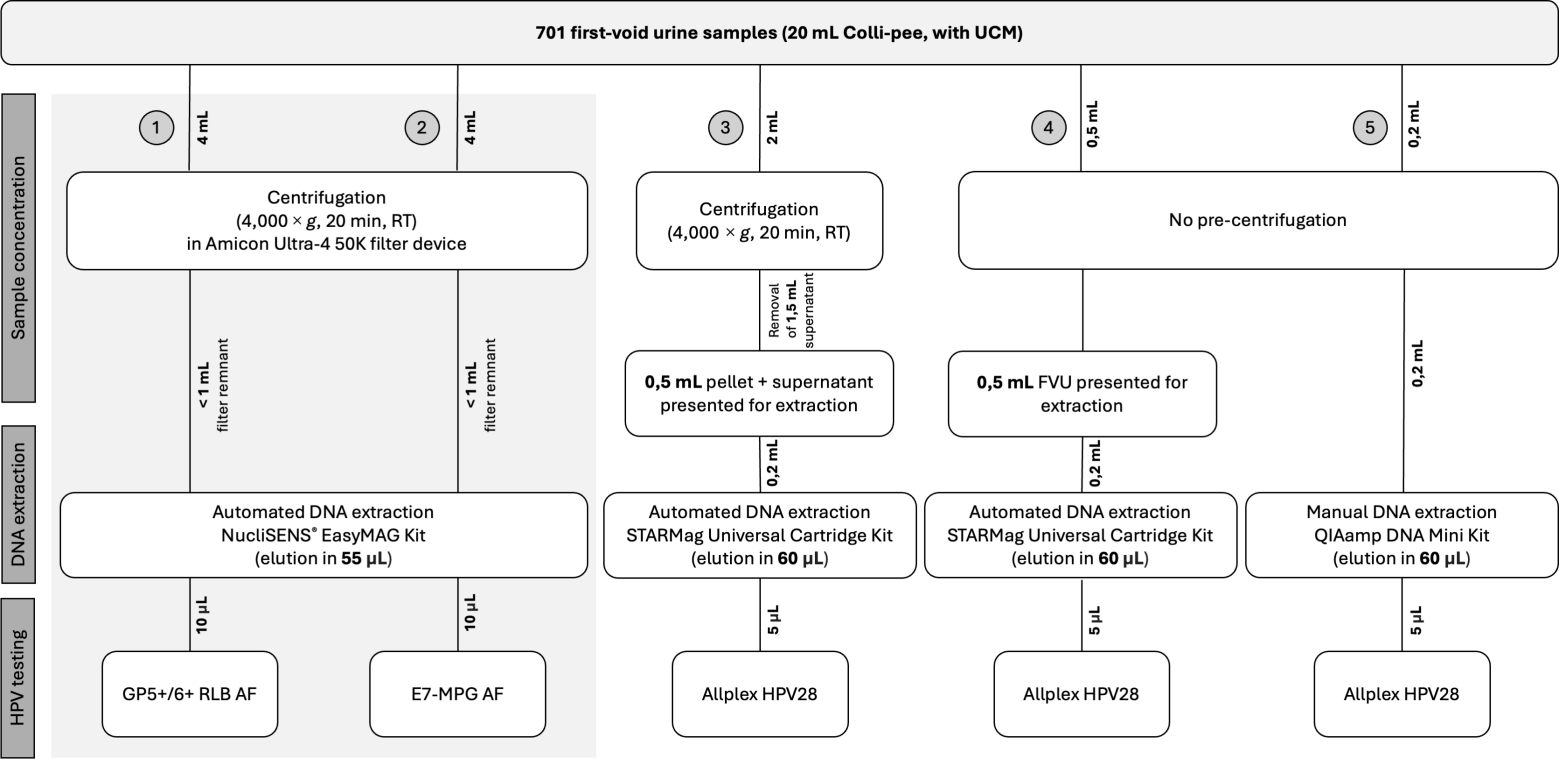
Schematic overview of the five different testing protocols that were used and compared within this study. AF, Amicon filtration; FVU, first-void urine; RT, room temperature; UCM, urine conservation medium.

Samples were shipped to the Centre for the Evaluation of Vaccinations, University of Antwerp, Belgium, where they were stored between −35°C and −80°C (Biobank Antwerpen, Antwerp, Belgium; ID: BE 710300310000) and tested blindly following five different testing protocols, as visualized in Fig. 1. In Arm 1 (historic), a combination of Amicon filtration (AF), NucliSENS EasyMag DNA extraction, and GP5+/6+ RLB testing was performed. In Arm 2 (historic), a combination of AF, NucliSENS EasyMag DNA extraction, and E7-MPG testing was performed. In Arm 3, samples were pre-centrifuged, DNA was extracted using the STARMag Universal Cartridge Kit, and Allplex HPV28 testing was performed. In Arm 4, samples were not pre-treated, DNA was extracted using the STARMag Universal Cartridge Kit, and Allplex HPV28 testing was performed. In Arm 5, samples were not pre-treated, DNA was extracted using the QIAamp DNA Mini Kit, and Allplex HPV28 testing was performed. Details of the different testing protocols are explained below.

### Sample concentration

#### Amicon filtration

For the AF, 4 mL of FVU was centrifuged at 4,000 × *g* for 20 min in an Amicon Ultra-4 50K filter device (Merck Millipore, Belgium) using a CF 48/-R centrifuge (Awel International, France). If after centrifugation, the volume on the filter remained >1 mL, centrifugation was repeated for 10 min until the starting volume for DNA extraction was <1 mL.

#### Pre-centrifugation

For the pre-centrifugation, 2 mL of FVU was centrifuged at 4,000 × *g* for 20 min, using a CF 48/-R centrifuge (Awel International, France). About 1.5 mL of the remaining volume was discarded, leaving a final volume of 0.5 mL to be loaded on the extraction device, which utilized 0.2 mL of the 0.5 mL for DNA extraction.

#### No pre-treatment

For the non-pretreated samples, 0.5 mL of FVU was directly loaded on the extraction device without prior centrifugation, with 0.2 mL of the 0.5 mL used for DNA extraction.

### DNA extraction

#### NucliSENS EasyMAG Kit

Automated DNA extraction using the NucliSENS EasyMag Kit (Bio-Mérieux, Benelux) was performed as reported elsewhere (13). Briefly, 2 mL of NucliSENS Lysis Buffer was added to the concentrate retained on the filter (<1 mL) and incubated for 10 min at room temperature. Subsequently, all materials were transferred to the NucliSENS Lysis buffer vial and DNA extraction was performed using the generic EasyMAG off-board lysis protocol, with an elution volume of 55 µL.

#### STARMag Universal Cartridge Kit

For the automated DNA extraction using the STARMag Universal Cartridge Kit (Seegene Inc., South Korea), 0.5 mL of FVU was presented to the STARlet device (Seegene Inc., South Korea), of which 0.2 mL was subjected to DNA extraction. Extraction was conducted in accordance with the manufacturer’s guidelines, following the Universal-2 protocol to elute DNA in a final volume of 60 µL.

#### QIAamp DNA Mini Kit (QIA)

Manual DNA extraction using the QIAamp DNA Mini Kit (Qiagen GmbH, Germany) was performed using 0.2 mL of FVU, following the manufacturer’s guidelines, with a final elution volume of 60 µL.

### HPV testing

#### Allplex HPV28

The Allplex HPV28 assay is an E6/E7/L1-based PCR-based assay, targeting 12 high-risk HPV genotypes (HPV16, HPV18, HPV31, HPV33, HPV35, HPV39, HPV45, HPV51, HPV52, HPV56, HPV58, and HPV59) as well as 16 other HPV genotypes (HPV6, HPV11, HPV26, HPV40, HPV42, HPV43, HPV44, HPV53, HPV54, HPV61, HPV66, HPV68, HPV69, HPV70, HPV73, and HPV82). The assay is based on a tagging oligonucleotide cleavage and extension technology, combined with a multiple detection temperature technology, allowing for the generation of individual cycle threshold (Ct) values for the 28 distinct genotypes at once. Using 5 µL of the DNA, Allplex HPV28 testing was performed at the Centre for the Evaluation of Vaccinations, University of Antwerp, Belgium, using the CFX96 real-time thermocycler (Bio-Rad, USA) following the manufacturer’s instructions (Ct cutoff 43.00). To confirm the presence of human cells, the assay targets the human beta-globin gene as internal quality control (Ct cutoff 43.00).

#### GP5+/6+ RLB

Using 10 µL of DNA, GP5+/6+ PCR EIA followed by RLB was performed at the Department of Pathology at the VU University Medical Center (currently known as Amsterdam UMC, location Vrije Universiteit), Amsterdam, the Netherlands, as described previously (13). Briefly, a combination of two oligoprobe cocktails was used to hybridize PCR products in an EIA, and EIA-positive samples were subsequently genotyped by RLB of the GP5+/6+ products (28), allowing for the genotype-specific detection of 44 mucosal HPV genotypes: HPV6, HPV11, HPV16, HPV18, HPV26, HPV30, HPV31, HPV32, HPV33, HPV34, HPV35, HPV39, HPV40, HPV42, HPV43, HPV44, HPV45, HPV51, HPV52, HPV53, HPV54, HPV55, HPV56, HPV57, HPV58, HPV59, HPV61, HPV64, HPV66, HPV67, HPV68, HPV69, HPV70, HPV71, HPV72, HPV73, HPV81, HPV82, HPV83, HPV84, HPV85, HPV86, HPV89, and HPV90, including all 28 genotypes detected by the Allplex HPV28. The GP5+/6+ PCR has previously been evaluated for its analytical sensitivity using the WHO HPV LabNet proficiency panel (32).

#### E7-MPG

Using 10 µL of DNA, E7-MPG testing was performed at IARC, Lyon, France, as described previously (13–15). Briefly, E7-MPG is a type-specific E7 PCR bead-based multiplex assay performed on a Luminex-based platform which is highly analytically sensitive to detect 12 high-risk HPV genotypes (HPV16, HPV18, HPV31, HPV33, HPV35, HPV39, HPV45, HPV51, HPV52, HPV56, HPV58, and HPV59) as well as 9 other HPV genotypes (HPV6, HPV11, HPV26, HPV53, HPV66, HPV68, HPV70, HPV73, and HPV82) (33). The following genotypes, that are detected by Allpex HPV28, are not detected by E7-MPG: HPV40, HPV42, HPV43, HPV44, HPV54, HPV61, and HPV69. Results are expressed as the median of the mean fluorescence intensity (MFI) of at least 100 beads per set, offering a semiquantitative measure of the number of copies of target DNA in the sample. The E7-MPG assay has previously been evaluated for its analytical sensitivity using the WHO HPV LabNet proficiency panel (32, 34).

### Statistical analysis

All statistical analyses were performed using R statistical software version 4.2.2. Genotype-specific positivity was calculated and compared graphically among valid (i.e., positive for the internal beta-globin control of the Allplex HPV28 assay) samples only. On graphs comparing genotype-specific positivity, a dotted line represents a theoretical scenario where both methods detect genotypes equally, and a solid line represents the slope of a linear regression passing through the origin and all genotype-specific points, that is, the average positivity ratio between compared methods, for which the strength of the association was assessed by using the coefficient of determination, *R*^2^, or explained variation. After assessing the normal distribution of variables (using the Shapiro–Wilk test and *Q–Q* plot), we conducted Bland-Altman and Spearman rank tests to study the association between Ct-values of both internal controls and HPV-positive samples for the different pre-treatment methods, and calculated 95% confidence intervals (95% CIs) using an equal tail percentage bootstrap (*n* = 1,000 repetitions). Furthermore, to assess genotype-specific agreement between the Allplex HPV28, GP5+/6+ RLB AF, and E7-MPG AF methods, percentage agreements, Cohen’s *κ* coefficients, and the corresponding 95% CIs were calculated (*κ*  ≤  0.20, poor; 0.21 ≤ *κ*  ≤  0.40, fair; 0.41 ≤ *κ*  ≤  0.60, moderate; 0.61 ≤ *κ*  ≤  0.80, good; and *κ*  ≥  0.81, excellent agreement). Mean Allplex HPV28 Ct-values and mean E7-MPG MFI-values were calculated for samples testing positive for two methods and samples testing positive for one of the two methods within the comparison, and Mann-Whitney *U* testing was performed to assess the significance of the difference between means.

## RESULTS

### Optimization of preanalytical methods

#### Pre-centrifugation versus direct automated DNA extraction

**Fig 2:**
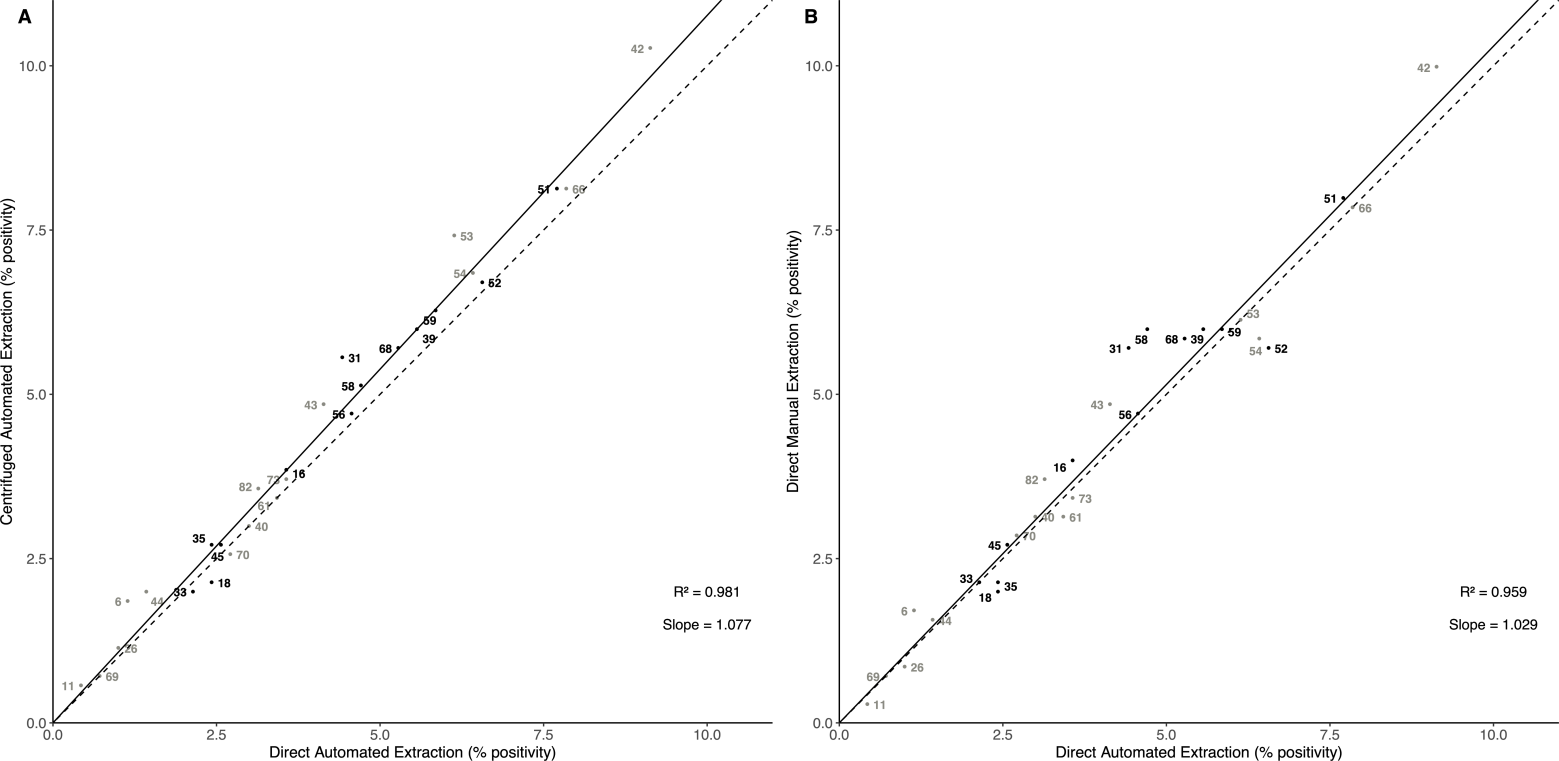
Comparison of genotype-specific positivity with Allplex HPV28 by direct automated DNA extraction versus (**A**) pre-centrifuged automated DNA extraction in 697^1^ FVU samples, and (**B**) direct manual DNA extraction in 691^2^ FVU samples. ^1^One sample tested invalid with Allplex HPV28 after pre-centrifuged automated DNA extraction, and three others after direct automated DNA extraction. ^2^Nine samples tested invalid after direct manual DNA extraction, of which two tested also invalid after direct automated DNA extraction. Dotted lines represent a theoretical scenario where genotypes are detected equally with both methods, and solid lines represent the slope of the linear regression passing through the origin and the 28 genotype-specific points. Black dots represent high-risk HPV genotypes, while gray dots represent other HPV genotypes.

Figure 2A compares genotype-specific positivity by Allplex HPV28 testing after pre-centrifuged versus direct DNA extraction. A strong comparability between the two methods was observed, with a high coefficient of determination (*R*^2^ = 0.981, *P* < 0.001), and slope of the regression line of 1.077 (*P* < 0.001). Percentage agreements were between 97.1% and 100% and kappa values were between 0.70 and 1.00, according to genotype, suggesting an almost perfect agreement between the two methods (Table S1).

When comparing Ct-values, significant Spearman rank correlations were found after pre-centrifugation versus direct DNA extraction, and Bland-Altman plots presented no systematic bias or outliers with increasing Ct-value, both for HPV-positive samples (Fig. S1) and internal beta-globin controls (Fig. S2).

#### Manual versus automated DNA extraction

Figure 2B compares genotype-specific positivity by Allplex HPV28 testing after manual versus automated DNA extraction, without pre-centrifugation. A strong comparability between the two methods was observed, with an *R*^2^ value of 0.959 and a slope of 1.029 (*P* < 0.001). High genotype-specific agreements were further supported by percentage agreements ranging from 95.7% to 100% and kappa values between 0.45 and 1.00 (Table S2). When comparing Ct-values, significant Spearman rank correlations were found after manual versus automated DNA extraction, and Bland-Altman plots presented no systematic bias or outliers with increasing Ct-value, both for HPV-positive samples (Fig. S3) and internal beta-globin controls (Fig. S4).

### Genotype-specific assay comparison

Given the strong agreement between the DNA extraction methods described above, we chose to compare the GP5+/6+ RLB and E7-MPG on Amicon filtrated and NucliSENS EasyMAG extracted samples (AF) with Allplex HPV28 results after direct automated DNA extraction. Out of 701 samples, 698 samples were valid (i.e., positive for the internal beta-globin control) with Allplex HPV28 after direct automated DNA extraction and were included in analyses comparing HPV genotyping tests.

#### Allplex HPV28 versus GP5+/6+ RLB AF

**Fig 3:**
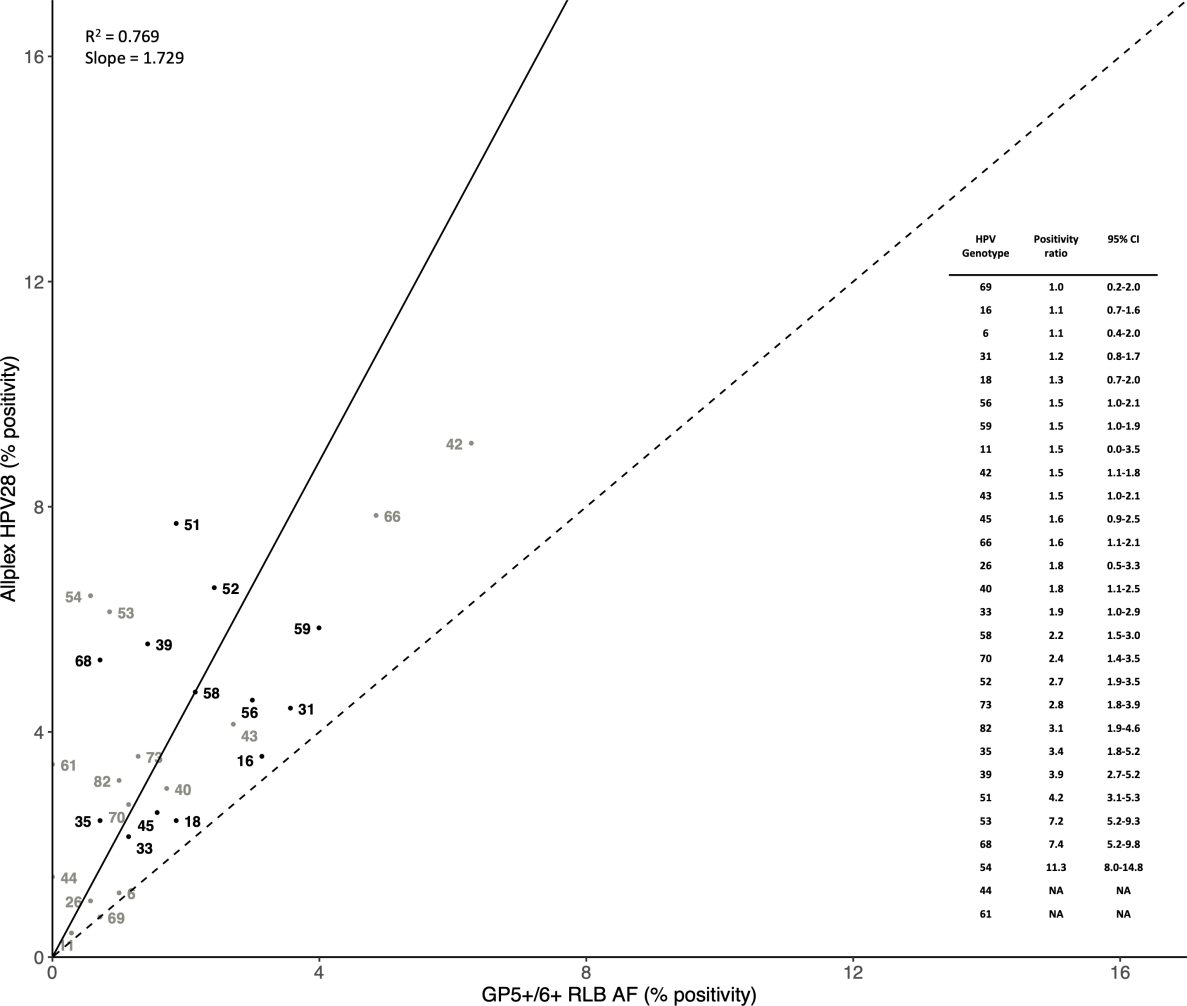
Comparison of genotype-specific positivity in 698 FVU samples by Allplex HPV28 (after direct STARMag Universal extraction) versus GP5+/6+ RLB (after Amicon filtration and NucliSENS EasyMAG extraction [AF]). The dotted line represents a theoretical scenario where genotypes are detected equally with both methods, and the solid line represents the slope of the linear regression passing through the origin and the 28 genotype-specific points. Black dots represent high-risk HPV genotypes, while gray dots represent other HPV genotypes.

With respect to the 28 HPV genotypes detected by both Allplex HPV28 and GP5+/6+ RLB AF, 266 (38.1%) FVU samples tested positive by both Allplex HPV28 and GP5+/6+ RLB AF, 100 (14.3%) by Allplex HPV28 alone, and 35 (5.0%) by GP5+/6+ RLB AF alone. Genotype-specific positivity by Allplex HPV28 and GP5+/6+ RLB AF is compared in Fig. 3. Linear regression analysis revealed an average Allplex HPV28:GP5+/6+ RLB AF ratio across the 28 detected HPV genotypes (i.e., the slope of the linear regression line) of 1.729, with an *R*^2^ value of 0.769 (*P* < 0.001), and positivity for all 28 HPV genotypes was higher by Allplex HPV28 than GP5+/6+ RLB AF (Allplex HPV28:GP5+/6+ RLB AF ratios all above 1.0). Ratios close to 1.0 were observed for quadrivalent vaccine types HPV6 (1.1), HPV11 (1.5), HPV16 (1.1), and HPV18 (1.3). The highest ratios were seen for HPV53 (7.2), HPV68 (7.4), and HPV54 (11.3).

Table 1 further describes genotype-specific correlation between Allplex HPV28 and GP5+/6+ RLB AF. Percentage agreements between the two methods ranged from 94.1% for HPV51 and HPV54, to 100% for HPV69, while kappa coefficients varied between 0 for HPV44 and HPV61 (no samples were positive with GP5+/6+ RLB AF) and 1.00 (95% CI = 1.00–1.00) for HPV69. Very few samples were positive for GP5+/6+ RLB AF but negative for Allplex HPV28. However, for all HPV genotypes, a number of samples were negative by GP5+/6+ RLB AF but positive for Allplex HPV28, of which a substantial proportion (50.0–100.0%) were positive also for E7-MPG AF, suggesting the true presence of infections at low viral loads. Furthermore, for most HPV genotypes, mean Allplex HPV28 Ct-values of infections detected by both Allplex HPV28 and GP5+/6+ RLB AF were significantly lower than infections detected only by Allplex HPV28, suggesting that GP5+/6+ RLB AF negativity was, at least in part, related to viral load.

**TABLE 1:** Genotype-specific comparison between Allplex HPV28 and GP5+/6+ RLB AF^*a*^

		Allplex HPV28/GP5+/6+ RLB AF results^*c*^							Mean Ct-value Allplex HPV28^*e*^	
Genotype^*b*^		–/–	–/+	+/–	+/+	% Agreement	Kappa coefficient (95% CI)	+/– samples pos. for E7-MPG AF (*n* (%))^*d*^	+/–	+/+
High-risk	HPV 16	672	1	4	21	99.3	0.89 (0.80–0.99)	2 (50.0)	40.63	30.17^*^
	HPV 18	681	0	4	13	99.4	0.86 (0.73–0.99)	4 (100.0)	38.80	29.72^*^
	HPV 31	667	0	6	25	99.1	0.89 (0.80–0.98)	6 (100.0)	38.35	29.79^*^
	HPV 33	683	0	7	8	99.0	0.69 (0.47–0.91)	7 (100.0)	35.39	30.12^*^
	HPV 35	681	0	12	5	98.3	0.45 (0.19–0.71)	11 (91.7)	35.00	28.18^*^
	HPV 39	659	0	29	10	95.8	0.39 (0.22–0.57)	28 (96.6)	33.23	25.74^*^
	HPV 45	680	0	7	11	99.0	0.74 (0.55–0.92)	5 (71.4)	39.79	30.57^*^
	HPV 51	644	0	41	13	94.1	0.37 (0.22–0.51)	40 (97.6)	34.22	26.19^*^
	HPV 52	652	0	29	17	95.8	0.52 (0.37–0.67)	29 (100.0)	35.69	28.96^*^
	HPV 56	666	0	11	21	98.4	0.79 (0.66–0.91)	11 (100.0)	39.84	30.64
	HPV 58	665	0	18	15	97.4	0.61 (0.45–0.78)	17 (94.4)	33.69	30.96
	HPV 59	657	0	13	28	98.1	0.80 (0.69–0.91)	11 (84.6)	38.88	30.54^*^
Other^*f*^	HPV 6	690	0	1	7	99.9	0.93 (0.80–1.00)	1 (100.0)	35.82	27.44
	HPV 11	695	0	1	2	99.9	0.79 (0.41–1.00)	1 (100.0)	38.66	27.16
	HPV 26	691	0	3	4	99.6	0.73 (0.43–1.00)	2 (66.7)	33.81	31.11
	HPV 40	677	0	9	12	98.7	0.72 (0.55–0.89)		35.17	27.69^*^
	HPV 42	632	2	22	42	96.5	0.76 (0.67–0.85)		35.49	28.69^*^
	HPV 43	668	1	11	18	98.3	0.74 (0.60–0.88)		36.13	29.18^*^
	HPV 44	688	0	10	0	98.6	0.00 (0.00–0.00)		33.93	NA
	HPV 53	655	0	37	6	94.7	0.20 (0.05–0.35)	28 (75.7)	34.08	26.37^*^
	HPV 54	653	0	41	4	94.1	0.15 (0.02–0.23)		33.84	32.04
	HPV 61	674	0	24	0	96.5	0.00 (0.00–0.00)		34.75	NA
	HPV 66	641	2	23	32	96.4	0.70 (0.59–0.81)	23 (100.0)	38.23	29.29^*^
	HPV 68	661	0	32	5	95.4	0.34 (0.06–0.39)	30 (93.8)	34.13	28.05^*^
	HPV 69	693	0	0	5	100.0	1.00 (1.00–1.00)		NA	34.39^*^
	HPV 70	679	0	11	8	98.4	0.59 (0.37–0.81)	9 (72.2)	36.79	29.99^*^
	HPV 73	673	0	16	9	97.7	0.52 (0.32–0.72)	16 (100.0)	35.18	26.32^*^
	HPV 82	676	0	15	7	97.9	0.44 (0.20–0.67)	14 (93.3)	34.26	26.03^*^

^*a*^ CI, confidence interval; NA, not applicable; Ct-value, cycle threshold value.

^*b*^ Twenty-eight HPV genotypes are detected by both Allplex HPV28 and GP5+/6+ RLB.

^*c*^ –/–, negative by both tests; –/+, positive only by GP5+/6+ RLB; +/–, positive only by Allplex HPV28; +/+, positive by both tests.

^*d*^ Shaded cells are HPV genotypes not detected by E7-MPG.

^*e*^ *, *P* < 0.05 for mean MFI of +/+ versus –/+.

^*f*^ Other HPV genotypes include possible high-risk, probable high-risk, and low-risk HPV genotypes.

#### Allplex HPV28 versus E7-MPG AF

**Fig 4:**
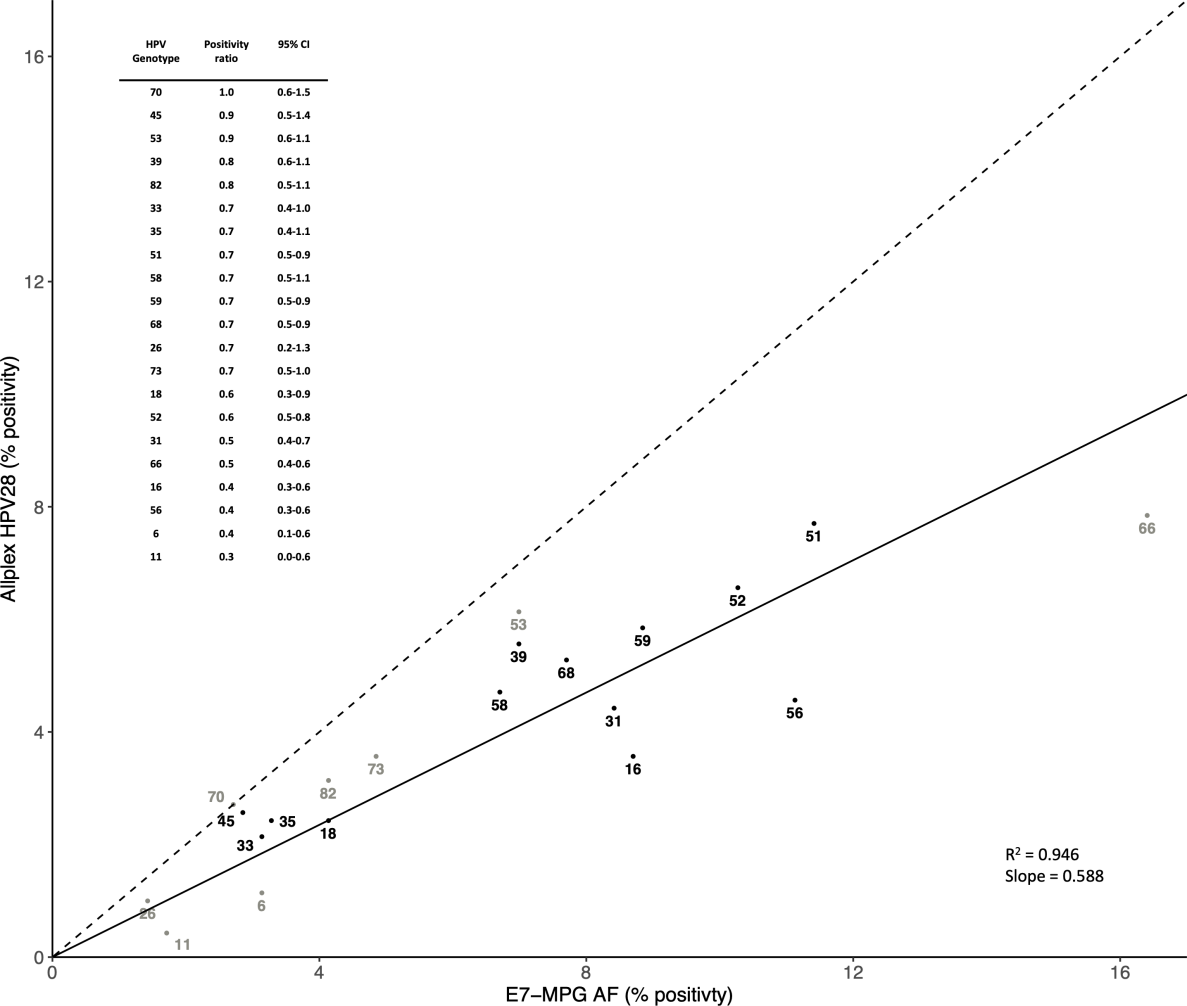
Comparison of genotype-specific positivity in 698 FVU samples by Allplex HPV28 (after direct STARMag Universal extraction) and E7-MPG (after Amicon filtration and NucliSENS EasyMAG extraction [AF]). The dotted line represents a theoretical scenario where genotypes are detected equally with both methods, and the solid line represents the slope of the linear regression passing through the origin and the 21 genotype-specific points. Black dots represent high-risk HPV genotypes, while gray dots represent other HPV genotypes.

With respect to the 21 HPV genotypes detected by both Allplex HPV28 and E7-MPG AF, 336 (48.1%) FVU samples tested positive by both methods, 30 (4.3%) by Allplex HPV28 only, and 238 (34.1%) by E7-MPG AF only. Genotype-specific positivity by Allplex HPV28 and E7-MPG AF is compared in Fig. 4. Linear regression analysis revealed an average Allplex HPV28:E7-MPG AF ratio across the 21 detected HPV genotypes (i.e., the slope of the linear regression line) of 0.588, with an *R*^2^ value of 0.946 (*P* < 0.001), and positivity for all 21 HPV genotypes was lower by Allplex HPV28 than E7-MPG AF (Allplex HPV28:E7-MPG AF ratios all equal to or below 1.0). Low ratios were observed for quadrivalent vaccine types HPV6 (0.4), HPV11 (0.3), HPV16 (0.4), and HPV18 (0.6). Highest ratios were seen for HPV70 (1.0), HPV45 (0.9), and HPV53 (0.9).

Table 2 further describes the genotype-specific correlation between Allplex HPV28 and E7-MPG AF. Percentage agreements between the two methods ranged from 91.5% for HPV66 to 99.3% for HPV26, with kappa values ranging from 0.40 (95% CI = 0.08–0.71) for HPV11 to 0.86 (95% CI = 0.77–0.94) for HPV39. Few samples were negative for E7-MPG AF but positive for Allplex HPV28, whereas there were many more samples negative for Allplex HPV28 and positive for E7-MPG AF (of which, apart from two samples for HPV66, no samples were positive for GP5/6+ RLB AF). For most HPV genotypes, mean E7-MPG AF MFI values of infections detected only by E7-MPG AF tended to be significantly higher among samples positive for the same genotype by Allplex HPV28, suggesting that negativity by Allplex HPV28 is related, at least in part, to a lower viral load. Notable exceptions were HPV35, HPV51, HPV26, HPV53, and HPV82.

**TABLE 2:** Genotype-specific comparison between the Allplex HPV28 and E7-MPG AF^*a*^

		Allplex HPV28/E7-MPG AF results^*d*^						Mean MFI-value E7-MPG AF^*e*^	
Genotype^*c*^		–/–	–/+	+/–	+/+	% Agreement	Kappa coefficient (95% CI)	–/+	+/+
High-risk	HPV 16	635	38	2	23	94.3	0.51 (0.38–0.64)	380.55	606.63^*^
	HPV 18	669	12	0	17	98.3	0.73 (0.59–0.88)	269.33	798.76^*^
	HPV 31	639	28	0	31	96.0	0.67 (0.56–0.78)	386.48	631.16^*^
	HPV 33	676	7	0	15	99.0	0.81 (0.67–0.95)	32.14	196.20^*^
	HPV 35	674	7	1	16	98.9	0.79 (0.66–0.93)	61.64	97.25
	HPV 39	648	11	1	38	98.3	0.86 (0.77–0.94)	34.14	167.05^*^
	HPV 45	675	5	3	15	98.9	0.78 (0.64–0.93)	25.10	121.37^*^
	HPV 51	617	27	1	53	95.9	0.76 (0.68–0.84)	116.94	125.79
	HPV 52	626	26	0	46	96.3	0.76 (0.67–0.85)	67.08	162.73^*^
	HPV 56	620	46	0	32	93.4	0.55 (0.44–0.66)	237.21	455.36^*^
	HPV 58	650	15	1	32	97.7	0.79 (0.69–0.89)	166.23	300.59^*^
	HPV 59	634	23	2	39	96.4	0.74 (0.64–0.84)	33.41	74.50^*^
Other^*f*^	HPV 6	675	15	1	7	97.7	0.46 (0.24–0.68)	25.87	74.07^*^
	HPV 11	686	9	0	3	98.7	0.40 (0.08–0.71)	81.67	803.83^*^
	HPV 26	687	4	1	6	99.3	0.70 (0.45–0.95)	368.50	172.00
	HPV 53	639	16	10	33	96.3	0.70 (0.59–0.81)	86.30	118.95
	HPV 66	584	59^*b*^	0	55	91.5	0.61 (0.52–0.70)	138.06	285.45^*^
	HPV 68	642	19	2	35	97.0	0.75 (0.65–0.86)	30.91	183.31^*^
	HPV 70	675	4	4	15	98.9	0.78 (0.64–0.93)	20.75	98.47^*^
	HPV 73	664	9	0	25	98.7	0.84 (0.74–0.94)	38.63	142.94^*^
	HPV 82	668	8	1	21	98.7	0.82 (0.70–0.93)	125.31	162.38

^*a*^ CI, confidence interval; MFI-value, mean fluorescence intensity value.

^*b*^ Apart from two samples for HPV66, no samples negative for Allplex HPV28 and positive for E7-MPG were positive for GP5+/6+ RLB.

^*c*^ Twenty-one HPV genotypes are detected by both Allplex HPV28 and E7-MPG.

^*d*^ –/–, negative by both tests; –/+, positive only by E7-MPG; +/–, positive only by Allplex HPV28; +/+, positive by both tests.

^*e*^ ^*^, *P* < 0.05 for mean MFI of +/+ versus –/+.

^*f*^ Other HPV genotypes include possible high-risk, probable high-risk, and low-risk HPV genotypes.

## DISCUSSION

Based on a highly informative set of previously genotyped FVU samples, this study established an analytical validation and optimization of pretreatment protocols of the Allplex HPV28 assay for HPV genotyping in FVU samples. Importantly, with respect to practical considerations for implementation of epidemiological studies around the world, HPV results were shown to be robust irrespective of whether automated or manual DNA extraction was used, and a pre-centrifugation step was proven unnecessary to obtain consistent results. Considering that GP5+/6+ PCR EIA has long been the benchmark for evaluating new HPV DNA tests for cervical cancer screening purposes, and, in combination with prior AF, has been used for long-term vaccine monitoring studies, our comparisons suggest that Allplex HPV28 can become an alternative for HPV genotyping in FVU samples.

Measuring HPV genotype-specific prevalence is essential for effective surveillance and monitoring of HPV vaccination, and FVU samples are particularly relevant to such work. The recent discontinuation of the GP5+/6+ RLB AF technique, long used within IARC’s vaccine monitoring study framework, underscores the need to validate alternative methods. Despite the potential of FVU as a credible specimen for HPV-related research and cervical cancer screening, the widespread implementation of urine-based HPV tests has been hindered by the lack of validated and commercially standardized HPV tests (35). As part of the VALHUDES framework and other validation experiments, a few HPV tests have been clinically validated for FVU (3, 4, 6–9), but none are capable of full quantitative genotyping required for vaccine impact monitoring. The ability of the Allplex HPV28 assay to individually detect 28 HPV genotypes with individual Ct-values, makes it a good option to adapt to FVU for HPV vaccination monitoring and epidemiologic research (30, 36), provided that the assay’s analytical characteristics remain consistent over time to allow for long-term monitoring of HPV prevalence.

No significant differences in the genotype-specific results by Allplex HPV28 were observed when comparing pre-centrifuged versus direct DNA extraction, affirming that pre-centrifugation of FVU samples prior to DNA extraction is unnecessary. Results of our comparison between manual versus automated DNA extraction of non-centrifuged samples before Allplex HPV28 testing also revealed an almost identical prevalence of all 28 HPV genotypes. These results align with other studies performed in cervical or anal samples, showing that pretreatment and DNA extraction methods have little or no impact on genotype-specific prevalence when using the Anyplex II HPV28 assay, the precursor of the Allplex HPV28 assay (37, 38).

Our findings hold significant relevance for real-world implementation, particularly in LMIC, where infrastructure and resources may be limited. Simplifying the DNA extraction process by omitting the centrifugation step not only reduces the risk of errors and contamination, but also minimizes hands-on work and lowers overall testing costs. Furthermore, in settings where automated DNA extraction is unavailable or unaffordable, manual DNA extraction presents an appealing and convenient alternative, while retaining a standardized protocol that still allows for pooling and comparison of multi-center studies detecting HPV from FVU.

Genotype-specific comparisons versus GP5+/6+ RLB AF showed systematically higher sensitivity for Allplex HPV28, with an average Allplex HPV28:GP5+/6+ RLB AF ratio for the 28 types of 1.7. Most samples testing positive by Allplex HPV28 but negative with GP5+/6+ RLB AF were positive with a third highly analytically sensitive method, E7-MPG AF, suggesting that these were not false positive Allplex HPV28 results. Rather, mean Allplex HPV28 Ct-values were generally lower for infections detected by both methods, suggesting that infections undetected by GP5+/6+ RLB AF were of lower viral copy number. In previous head-to-head clinical validations in cervical cells, Anyplex II (the predecessor of Allplex) was also shown to have slightly higher detection of high-risk genotypes than GP5+/6+ PCR (39) and Hybrid Capture 2 (40–42), even if clinical specificity for <CIN2 was considered to be statistically non-inferior (43).

The size differences in sensitivity between Allplex HPV28 and GP5+/6+ RLB AF varied by genotype. Lower agreements and higher Allplex HPV28:GP5+/6+ RLB AF ratios were observed for types such as HPV53, HPV68, and HPV54, closely matching those genotypes that revealed lower GP5+/6+ RLB detection rates also in previous studies (15, 44, 45). Moreover, also consistent with previous findings (15) HPV16 and HPV18, the two most carcinogenic HPV genotypes, exhibited high agreements between the two methods.

HPV tests that are more clinically specific (such as GP5+/6+ RLB) and those that are highly analytically sensitive (such as E7-MPG) are expected to behave differently when monitoring the impact of HPV vaccination in FVU. The use of highly sensitive assays such as E7-MPG (11, 13–15) may improve statistical power, particularly for rare HPV genotypes. Furthermore, the concentration of HPV DNA found in FVU can be lower (46), so employing methods with enhanced analytical sensitivity may be useful to standardize the assessment of vaccine-induced changes (47). On the other hand, estimates of vaccine effectiveness against HPV6/11/16/18 have been shown to be lower using more sensitive assays (11, 13), likely due to increased detection of low-copy HPV DNA with no clinical significance. Being slightly more sensitive as compared to the GP5+/6+ RLB AF, but clearly not as sensitive as E7-MPG AF, Allplex HPV28 on FVU samples without pre-concentration appears to offer a good alternative for detecting HPV in FVU to monitor vaccine impact.

It is important to note that the HPV assays within this study were performed on DNA extracts obtained through different pretreatment and DNA extraction methods. This approach aimed to streamline the workflow for real-world implementation by optimizing the complete methodology rather than focusing solely on the HPV assays themselves. However, this strategy introduces a discrepancy in the proportion of the original material used for final testing (Table S3), complicating the determination of whether the higher prevalence measured by E7-MPG is attributable purely to its higher analytical sensitivity or also to the larger amount of input material used. Additionally, despite the high overall HPV positivity in our sample set, a limitation of our study is the underrepresentation of certain HPV genotypes, potentially affecting the generalizability of our findings.

Further work may establish genotype-specific Ct-cutoffs for Allplex HPV28 in FVU based on clinical endpoints, as recently done for cervical cells (36). Finally, the high sample validity of the FVU samples, which were stored for up to 10 years, proves the stability of the urine sample matrix, allowing the possibility of retrospective testing of historical samples to maximize comparability across repeated prevalence surveys in the future.

### Conclusion

The findings of this study, combined with practical considerations for real-world implementation, support the use of Allplex HPV28 testing after either automated or manual DNA extraction without requirement for pre-centrifugation, for HPV studies based on FVU samples, most notably those for vaccine impact monitoring on HPV prevalence.

## Data Availability

The data presented in this study are available from the corresponding author upon reasonable request.

## SUPPLEMENTAL MATERIAL

Supplemental material jcm.01404-24-s0001.docx Figures S1 to S4; Tables S1 to S3.

ASM does not own the copyrights to Supplemental Material that may be linked to, or accessed through, an article. The authors have granted ASM a non-exclusive, world-wide license to publish the Supplemental Material files. Please contact the corresponding author directly for reuse.
