# Supplementary material for: Optimization and analytical validation of the Allplex HPV28 genotyping assay for use in first-void urine samples
Source: J Clin Microbiol. 2024 Dec 26;63(2):e01404-24. doi: 10.1128/jcm.01404-24 (PMC11837537; doi:10.1128/jcm.01404-24)
Supplement: Supplemental material — Figures S1 to S4; Tables S1 to S3. [file jcm.01404-24-s0001.docx]

**Supplementary material**

| **Table S1. Genotype-specific comparison of Allplex HPV28 testing after direct automated versus centrifuged automated DNA extraction.** | | | | | | | |
| --- | --- | --- | --- | --- | --- | --- | --- |
|  | | Allplex HPV28 results by direct and centrifuged DNA extraction^b^ | | | |  |  |
| Genotype^a^ | | –/– | –/+ | +/– | +/+ | % Agreement | Kappa coefficient (95% CI) |
| High-risk | HPV 16 | 667 | 3 | 5 | 22 | 98,9 | 0.84 (0.73-0.95) |
|  | HPV 18 | 679 | 3 | 1 | 14 | 99,4 | 0.87 (0.75-0.99) |
|  | HPV 31 | 658 | 0 | 8 | 31 | 98,9 | 0.88 (0.79-0.96) |
|  | HPV 33 | 681 | 2 | 1 | 13 | 99,6 | 0.89 (0.78-1.00) |
|  | HPV 35 | 677 | 1 | 3 | 16 | 99,5 | 0.89 (0.78-0.99) |
|  | HPV 39 | 677 | 1 | 4 | 38 | 99,3 | 0.93 (0.88-0.99) |
|  | HPV 45 | 675 | 3 | 4 | 15 | 98,9 | 0.81 (0.67-0.95) |
|  | HPV 51 | 637 | 3 | 6 | 51 | 98,7 | 0.91 (0.86-0.97) |
|  | HPV 52 | 647 | 3 | 4 | 43 | 98,9 | 0.92 (0.86-0.98) |
|  | HPV 56 | 662 | 2 | 3 | 30 | 99,3 | 0.93 (0.85-0.99) |
|  | HPV 58 | 658 | 3 | 6 | 30 | 98,7 | 0.86 (0.77-0.95) |
|  | HPV 59 | 651 | 2 | 5 | 39 | 98,9 | 0.91 (0.85-0.98) |
| Other^c^ | HPV 6 | 684 | 0 | 5 | 8 | 99,3 | 0.70 (0.55-0.96) |
|  | HPV 11 | 693 | 0 | 1 | 3 | 99,9 | 0.86 (0.58-1.00) |
|  | HPV 26 | 688 | 1 | 2 | 6 | 99,6 | 0.79 (0.57-1.00) |
|  | HPV 40 | 675 | 1 | 1 | 20 | 99,7 | 0.95 (0.88-1.00) |
|  | HPV 42 | 623 | 2 | 10 | 62 | 98,3 | 0.90 (0.85-0.96) |
|  | HPV 43 | 661 | 2 | 7 | 27 | 98,7 | 0.85 (0.75-0.95) |
|  | HPV 44 | 683 | 0 | 4 | 10 | 99,4 | 0.83 (0.67-0.99) |
|  | HPV 53 | 643 | 2 | 11 | 41 | 98,1 | 0.85 (0.78-0.93) |
|  | HPV 54 | 647 | 2 | 5 | 43 | 98,9 | 0.92 (0.86-0.98) |
|  | HPV 61 | 671 | 2 | 2 | 22 | 99,4 | 0.91 (0.83-0.99) |
|  | HPV 66 | 632 | 9 | 11 | 45 | 97,1 | 0.80 (0.72-0.89) |
|  | HPV 68 | 654 | 3 | 6 | 34 | 98,7 | 0.88 (0.79-0.96) |
|  | HPV 69 | 692 | 0 | 0 | 5 | 100,0 | 1.00 (1.00-1.00) |
|  | HPV 70 | 676 | 3 | 2 | 16 | 99,3 | 0.86 (0.74-0.98) |
|  | HPV 73 | 669 | 2 | 3 | 23 | 99,6 | 0.94 (0.87-1.00) |
|  | HPV 82 | 672 | 0 | 3 | 22 | 99,6 | 0.93 (0.86-1.00) |
| CI, confidence interval.  ^a^28 HPV genotypes are detected by Allplex HPV28.  ^b^–/–, negative by both; –/+, positive only after centrifugation; +/–, positive only after direct testing; +/+, positive by both.  ^c^Other HPV genotypes include possible high-risk, probable high-risk, and low-risk HPV genotypes. | | | | | | | |

**Figure S1. Comparison between Ct-values of HPV-positive samples using Allplex HPV28 testing after pre-centrifuged automated DNA extraction versus direct automated DNA extraction, visualized in a (A) Spearman correlation plot and (B) Bland-Altman plot.** On the Spearman correlation plot, the Spearman rank correlation coefficient (r) with 95% confidence interval and p-value is indicated in the figure, and the red dotted line represents a theoretical scenario where Ct-values are equal with both methods. On the Bland-Altman plot, the mean of the differences between both methods is represented in red, while the upper and lower black dotted lines represent the mean plus and minus 1.96 times the standard deviation (SD) of the differences, respectively.

**

**Figure S2.** **Comparison between Ct-values of internal beta-globin controls using Allplex HPV28 testing after pre-centrifuged automated DNA extraction versus direct automated DNA extraction, visualized in a (A) Spearman correlation plot and (B) Bland-Altman plot.** On the Spearman correlation plot, the Spearman rank correlation coefficient (r) with 95% confidence interval and p-value is indicated in the figure, and the red dotted line represents a theoretical scenario where Ct-values are equal with both methods. On the Bland-Altman plot, the mean of the differences between both methods is represented in red, while the upper and lower black dotted lines represent the mean plus and minus 1.96 times the standard deviation (SD) of the differences, respectively.

| **Table S2. Genotype-specific comparison between manual versus automated DNA extraction and Allplex HPV28 testing.** | | | | | | | |
| --- | --- | --- | --- | --- | --- | --- | --- |
|  | | No. (%) of samples by manual Allplex HPV28  and automated Allplex HPV28 results^b^ | | | |  |  |
| Genotype^a^ | | –/– | –/+ | +/– | +/+ | % Agreement | Kappa coefficient (95% CI) |
| High-risk | HPV 16 | 655 | 8 | 17 | 11 | 97,3 | 0.45 (0.27-0.63) |
|  | HPV 18 | 671 | 6 | 3 | 11 | 98,7 | 0.70 (0.52-0.89) |
|  | HPV 31 | 646 | 5 | 14 | 26 | 97,3 | 0.72 (0.59-0.84) |
|  | HPV 33 | 673 | 3 | 3 | 12 | 99,1 | 0.80 (0.64-0.96) |
|  | HPV 35 | 673 | 3 | 1 | 14 | 99,4 | 0.87 (0.75-0.99) |
|  | HPV 39 | 642 | 7 | 10 | 32 | 97,6 | 0.78 (0.68-0.88) |
|  | HPV 45 | 667 | 5 | 6 | 13 | 98,4 | 0.70 (0.52-0.87) |
|  | HPV 51 | 625 | 10 | 12 | 44 | 96,8 | 0.78 (0.70-0.87) |
|  | HPV 52 | 637 | 14 | 8 | 32 | 96,9 | 0.73 (0.62-0.84) |
|  | HPV 56 | 649 | 9 | 10 | 23 | 97,3 | 0.70 (0.56-0.82) |
|  | HPV 58 | 644 | 5 | 14 | 28 | 97,3 | 0.73 (0.62-0.85) |
|  | HPV 59 | 640 | 9 | 10 | 32 | 97,3 | 0.76 (0.65-0.86) |
| Other^c^ | HPV 6 | 676 | 3 | 7 | 5 | 98,6 | 0.49 (0.22-0.76) |
|  | HPV 11 | 687 | 2 | 1 | 1 | 99,6 | 0.63 (0.47-0.78) |
|  | HPV 26 | 684 | 1 | 0 | 6 | 99,9 | 0.92 (0.77-1.00) |
|  | HPV 40 | 666 | 3 | 4 | 18 | 99,0 | 0.83 (0.71-0.95) |
|  | HPV 42 | 610 | 12 | 18 | 51 | 95,7 | 0.75 (0.67-0.84) |
|  | HPV 43 | 651 | 6 | 11 | 23 | 97,6 | 0.72 (0.59-0.85) |
|  | HPV 44 | 677 | 3 | 4 | 7 | 99,0 | 0.66 (0.43-0.89) |
|  | HPV 53 | 635 | 13 | 14 | 29 | 96,1 | 0.66 (0.54-0.78) |
|  | HPV 54 | 642 | 8 | 4 | 37 | 98,3 | 0.85 (0.77-0.93) |
|  | HPV 61 | 665 | 4 | 2 | 20 | 99,1 | 0.87 (0.76-0.97) |
|  | HPV 66 | 621 | 15 | 15 | 40 | 95,7 | 0.70 (0.60-0.80) |
|  | HPV 68 | 647 | 4 | 7 | 33 | 98,4 | 0.85 (0.76-0.94) |
|  | HPV 69 | 686 | 0 | 0 | 5 | 100,0 | 1.00 (1.00-1.00) |
|  | HPV 70 | 666 | 5 | 6 | 14 | 98,4 | 0.71 (0.55-0.87) |
|  | HPV 73 | 662 | 5 | 5 | 19 | 99,6 | 0.77 (0.63-0.90) |
|  | HPV 82 | 662 | 3 | 7 | 19 | 98,6 | 0.78 (0.65-0.91) |
| CI, confidence interval.  ^a^28 HPV genotypes are detected by Allplex HPV28.  ^b^–/–, negative by both; –/+, positive only after automated extraction; +/–, positive only after manual extraction; +/+, positive by both.  ^c^Other HPV genotypes include possible high-risk, probable high-risk, and low-risk HPV genotypes. | | | | | | | |

**Figure S3**. **Comparison between Ct-values of HPV-positive samples using Allplex HPV28 testing after direct automated DNA extraction versus direct manual DNA extraction, visualized in a (A) Spearman correlation plot and (B) Bland-Altman plot.** On the Spearman correlation plot, the Spearman rank correlation coefficient (r) with 95% confidence interval and p-value is indicated in the figure, and the red dotted line represents a theoretical scenario where Ct-values are equal with both methods. On the Bland-Altman plot, the mean of the differences between both methods is represented in red, while the upper and lower black dotted lines represent the mean plus and minus 1.96 times the standard deviation (SD) of the differences, respectively.

**Figure S4. Comparison between Ct-values of internal beta-globin controls using Allplex HPV28 testing after direct automated DNA extraction versus direct manual DNA extraction, visualized in a (A) Spearman correlation plot and (B) Bland-Altman plot.** On the Spearman correlation plot, the Spearman rank correlation coefficient (r) with 95% confidence interval and p-value is indicated in the figure, and the red dotted line represents a theoretical scenario where Ct-values are equal with both methods. On the Bland-Altman plot, the mean of the differences between both methods is represented in red, while the upper and lower black dotted lines represent the mean plus and minus 1.96 times the standard deviation (SD) of the differences, respectively.

| **Table S3. Calculations of final volumes used for HPV testing, based on the sample testing flow presented in Figure 1.** | | |
| --- | --- | --- |
| Study arm | Calculation | Final volume  used for HPV testing |
| **Arm 1** | $\frac{4000 \mu L x 10 \mu L}{55 \mu L}$ | 727.3 μL |
| **Arm 2** | $\frac{4000 \mu L x 10 \mu L}{55 \mu L}$ | 727.3 μL |
| **Arm 3** | $\frac{2000 \mu L x 200 \mu L x 5 \mu L}{500 \mu L x 60 \mu L}$ | 16.7-66.7 μL^*^ |
| **Arm 4** | $\frac{200 \mu L x 5 \mu L}{60 \mu L}$ | 16.7 μL |
| **Arm 5** | $\frac{200 \mu L x 5 \mu L}{60 \mu L}$ | 16.7 μL |
| ^*^Depending on amount of DNA in pellet or supernatant after centrifugation. | | |
